# The impact of government macroeconomic policies and elections on income smoothing in state-controlled commercial banks: Evidence from the MENA region

**DOI:** 10.1371/journal.pone.0343259

**Published:** 2026-04-13

**Authors:** Pejman Peykani, Mostafa Sargolzaei, Mohammad Javad Mohagheghnia, Amir Takaloo, Mahsa Valimoradi

**Affiliations:** 1 Department of Industrial Engineering, Faculty of Engineering, Khatam University, Tehran, Iran; 2 Department of Finance and Banking, Faculty of Management and Accounting, Allameh Tabataba’i University, Tehran, Iran; Institute of Economics (Scuola Superiore Sant'Anna / RFF-CMCC European Institute on Economics and the Environment, ITALY

## Abstract

In most of the economies of different countries in the world, banks play a very important role in economic stability. We know that if the banks are not managed correctly, this issue will have heavy economic, political and social consequences for the society of that country. If we can find the important factors in the correct and accurate management of banks in the economy of a country, it will help the sustainable economic growth of that economy. In this research, we seek to find out how political shareholders affect the management of banks. To achieve this goal, the financial information of commercial banks of ten countries in the Middle East and North Africa (MENA) region during the years 2015–2020 and also the Generalized Method of Moments (GMM) model have been used. After calculating the percentage of government shares in the investigated banks, we analyze income smoothing using loan loss provisions (LLPs). One of the factors that has been considered in this regard is the elections in these countries and income smoothing in election years has been compared with other years. The obtained results show that income smoothing is more in banks that have more government shareholders. Another noteworthy point is that the election factor has a significant impact on the management of banks.

## 1. Introduction

The result of the correct management of banks shows itself in the financial statements and according to capitalists, highly fluctuating income is a sign of the high risk of financial institutions [1–7]. Therefore, one of the motivations of managers to reduce income fluctuations by using income smoothing is to gain the trust of bank shareholders. Bank managers use loan loss provisions (LLPs) to smooth out income because LLPs had an optional amount in addition to the legal amount that is calculated differently in each country, which bank managers can consider as a reserve based on their view of possible loan losses. As a result, there is a positive relationship between LLPs and income before tax and LLPs. The higher the income, the higher the LLPs, and the lower the income, the lower the LLPs avoid reducing the transparency of financial statements, regulatory rules and regulations have been implemented, usually governments or organizations related to the government are responsible for monitoring the correct implementation of laws. In a research on the effect of LLPs on stock value, it has been stated that the motivation to increase the stock value was the reason for managers to use LLPs to smooth income [8–19].

The tax reform laws of 1986, which were intended to prevent the behavior of income smoothing, did not have an effect, and banks use LLPs to smooth income, and this behavior is acceptable from the point of view of the stock market [20–22].

It has been stated that in companies with centralized ownership, there is a conflict of interest between the owners and foreign investors, as a result of this conflict, the owners control the company’s financial reports and reduce financial transparency, and the reported profit for investors is not real and Income smoothing takes place [13,23–30].

The major shareholders of the bank have the power to interfere in the way resources are managed and change the financial statements. There is little research on how the relationship between shareholders and income smoothing behavior. Among the major shareholders, the government shareholders who also have political relations are more likely to reduce financial transparency. The political goals of these shareholders lead to their dominance over the minority shareholders [31–37]. Also, government intervention and lack of proper supervision are weak factors of bank lending operations, and the need to use international accounting standards and international laws is emphasized in developing economies [38,39].

In another research, Sapienza [40] show the influence of the government on lending operations in Italy was investigated and it was stated that the state banks received lower interest rates under the influence of political factors and in the election years, the state banks gave their loans to the state-owned companies. The political party itself has given less interest and the managers have smoothed the income by using LLPs to eliminate the effects of these loans. The difference between the performance of public and private banks in developing countries during the election year is proof of the support of public banks for political views, also the performance of public banks has been weaker and despite higher costs, they have had lower income [8,9,41–45].

By examining the difference in income smoothing through LLPs in developing and developed countries, it has been shown that in developing countries, the percentage of shares was higher than in the hands of the government and politicians, and the government was involved in the distribution of banking resources. Weakness in auditing and financial transparency have been other effective factors in smoothing the income of banks [41,46–50].

The purpose of this paper is to examine whether banks have engaged in income smoothing through LLPs. In particular, we examine whether banks with government shareholders have a political incentive to smooth income. To this end, we use elections as a political factor to examine the effect of public shareholders on income smoothing.

In this research, we have examined all banks without considering the percentage of government shares. In recent years, policies have been implemented in developing countries to privatize banks. So, we first examined the shareholders and found the percentage of government shares in banks and classified these banks based on their percentage of government shares. In previous studies, the influence of government shareholders on state-owned banks has been discussed, here the effect of the policy factor on all banks in addition to state-owned banks has been investigated.

To discover the relationship between political goals and income smoothing, we consider the national election factor as a political goal and check whether this political goal will increase income smoothing or not. We consider the election year as the period in which political goals are higher than the average [51].

Some studies show evidence of government-controlled banks supporting political goals. But in this article, we examine all banks regardless of the percentage of government shares and whether they are under government control to discover whether only government-controlled banks are affected by political goals or all banks support political goals to gain political trust.

One of the central assumptions in this study is that political cycles, particularly election years, can influence how banks—especially those with government shareholders—manage their financial reporting. In countries with high state ownership in the banking sector, managers of public banks may face implicit political pressure to show financial stability during election periods. This is especially true when incumbent governments aim to demonstrate economic strength to the public. As a result, managers may use income-smoothing strategies, such as adjusting LLPs, to present consistent profitability and avoid drawing attention to any volatility in performance.

Moreover, bank managers themselves may have personal incentives to maintain or improve the financial appearance of their institutions in election years. In many developing economies, changes in political leadership often lead to turnover in senior management of state-owned banks. Thus, by showing better financial results, managers may seek to secure their positions and avoid being replaced after elections. This behavioral channel further strengthens the expectation that income smoothing will intensify in public banks during politically sensitive periods. These theoretical considerations underpin our focus on election years as a proxy for political influence and motivate the interaction terms used in our empirical models.

Based on the above, we posit that governments may have implicit incentives to encourage stable and favorable financial reporting from banks in election years. In particular, when governments are shareholders in banks, either directly or indirectly through state-owned institutions, they may prefer not to show financial volatility that could weaken public trust during political campaigns. Bank managers, under such implicit expectations, may use LLPs more aggressively to smooth income and present a stable financial image. This theoretical link between political cycles, government ownership, and income smoothing provides the rationale for using national election years as a proxy for political influence in this study.

We use an international bank sample from the Bank Scope database with 625 annual bank observations in ten Middle East and North Africa (MENA) countries over the period 2015–2020. The results of these observations show that despite the privatization policies announced by the governments, the governments are still part of the shareholders of the banks through their subsidiaries. Among bank shareholders, income smoothing has been done in all banks regardless of whether they are government or private. But in banks that have a higher percentage of government shares, the level of income smoothing has been higher, especially in the election year, these banks experience the highest level of income smoothing, which shows the impact of political policies and goals on income smoothing.

Today, monetary and banking organizations play an important role in people’s life cycle as well as economic infrastructure, so providing services based on open banking and also based on different channels can create different user experiences for people. On the other hand, banks’ investment in the country’s infrastructural affairs, including the development of housing and factories, urban modernization and providing various facilities based on the needs of society, can provide all-round growth and highlight the strong role of these institutions as operational arms of the economy.

Undoubtedly, MENA banks develop their services based on the software and hardware infrastructure provided by IT companies. This cooperation has made companies and banks dependent on each other like a tangled web. One of the challenges of banks in the MENA area is the allocation of appropriate currency and in the optimal timing, in this context, adjusting customs laws and speeding up the processes for the purpose of clearance of goods is very important. On the other hand, it is expected that companies that facilitate the entry of new technology into MENA countries, provide modern services to different sections of the society, so that they can enjoy appropriate tax incentives in this area. Also, what is called modern service tools in the banking system of the MENA region today, is far from similar tools in the world, especially tools in the field of validation and provision of facilities.

The remainder of this paper is organized as follows. In Section 2, it has been tried to refer to the researches that have been done in the past about income smoothing in banks and LLPs. Section 3 sets up the basic models and the estimating method. Section 4 displays the data description and result of the method. Section 5 Discuss the results of the paper and examine the effect of each independent variable on the LLPs. In section 6 the results of the models are summarized.

## 2. Literature review

In this section, a review of applied research is presented about ALM of banks using different models.

**Fig 1:**
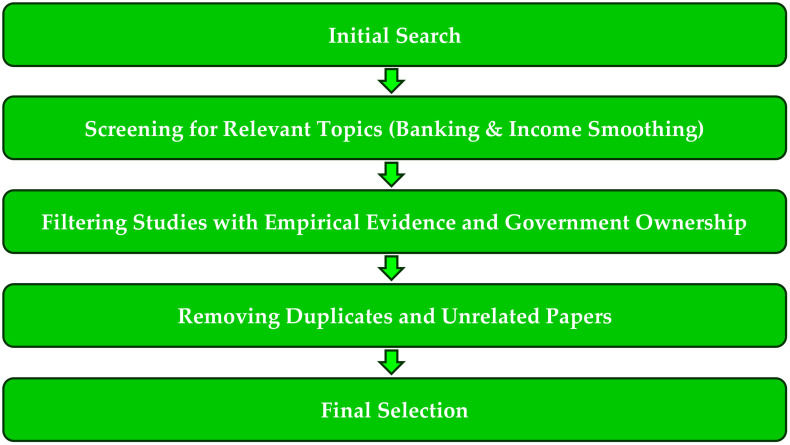
The PRISMA guidelines.

To conduct a systematic literature review (SLR) in alignment with the PRISMA framework, we followed a structured approach to selecting relevant studies. First, we searched multiple databases (e.g., Scopus, ScienceDirect, and Google Scholar) using keywords such as “bank income smoothing,” “loan loss provisions,” and “financial regulation.” Next, we applied inclusion criteria, selecting only studies that examined the relationship between LLPs and income smoothing in banks. In the third step, we further refined our selection by focusing on research that also analyzed the impact of government ownership on income smoothing. After removing duplicates and excluding papers that lacked empirical evidence or were unrelated, we finalized these studies for detailed analysis. The selection process is depicted in Fig 1, following PRISMA guidelines.

Income smoothing is an action in line with the use of accounting methods to smooth the fluctuations of net income in different reporting periods. If income smoothing is subject to generally accepted accounting principles (GAAP), the process will not be illegal. The income smoothing process involves the transfer of income and expenses from one accounting period to another. Reasons for income smoothing include tax reduction, attracting investors and as one of the components of business strategy.

The purpose of income smoothing is to reduce the fluctuations in income from one period to another to make the company appear to have stable income. The goal of this income smoothing procedure is related to high-income periods versus low-income periods or high-cost periods versus low-cost periods. Accountants do this by moving revenues and expenses in a legal way.

Examples of income smoothing techniques include deferring revenue recognition during one year when the following year is expected to be a challenging year or delaying the recognition of expenses in a difficult year when company performance is expected to improve in the near future.

Loan loss provision is used to predict the lending losses of banks. One of the most important tasks of banks around the world is to lend to individuals and small and large companies, and in this way they earn significant income. However, since the banking crisis of 2008, lending laws and the way banks operate have undergone serious changes, and lending standards and the quality of validation methods have greatly increased, reducing liquidity risk in banks. However, banks calculate their default risk.

Bhat [21] has sought to investigate income smoothing in banks using the financial information of these banks during the years 1981–1991. The purpose of this research was to analyze income smoothing using LLPs. The obtained results show that in banks where income smoothing is done using LLPs, after tax deduction, they seek profit smoothing and this relationship is significant. It is also evident from the results that the tax reform law of 1986 has not been able to have a special effect on income smoothing. Among the factors that affect profit smoothing, we can mention low book-to-asset ratio, high loans-to-deposit ratio, high debt-to-asset ratio, low market-to-book value ratio, low return on asset and this applies to banks with low growth.

In a research, Fonseca & Gonzalez [50] sought to determine what factors affect income smoothing using LLPs and to achieve this goal, he used the financial information of banks from 40 countries around the world. The obtained results show that among the factors that affect income smoothing using LLPs, it can be mentioned investor protection, disclosure, regulation and supervision, financial structure and financial development that income smoothing with investor protection, disclosure, Regulations and supervision have a negative relationship and a positive relationship with market orientation and financial development.

We know that banks make different decisions about LLPs according to their different motivations. In a research, Ozili [49] has sought to examine these different motivations and determine how LLPs are affected by these conditions. When Nigerian banks voluntarily implemented IFRS, signs of income smoothing were seen in these banks. After the implementation of this standard and the examination of these conditions by this research, the obtained results show that LLPs have increased in the investigated banks and the motivations discussed cause them to change LLPs and according to different conditions, the decision of bank managers to the choice between capital management and income smoothing is different. Therefore, according to the results, it is evident that the implementation of IFRS standard increases the motivation of banks to change LLPs.

Vasilakopoulos [18] has tried to examine the relationship between governance mechanisms and income smoothing and its impact on managers’ policies regarding LLPs in European banks. To achieve this goal, the financial information of 98 banks from 23 European countries during the years 2010–2013 and a multivariate regression model were used. The obtained results show that the relationship between LLPs and income smoothing is significant and positive and that income smoothing follows a specific pattern. Also, a significant point that is evident in the results is that managers make different decisions to smooth the income, according to different reporting conditions.

Ozili [11], in a research, sought to investigate how income smoothing using LLPs affects IAS 39 reclassification. The idea in this research is that the income smoothing of banks is reduced due to the reclassification of IAS 39 and the use of bank securities and derivatives and it should be done using LLPs, which rejects this idea. Also, the results show that income smoothing using LLPs has not been done by European banks.

Pinto et al. [13] has tried to analyze the impact of corporate governance and foreign investment on income smoothing using LLPs in African banks. To achieve the goal of this research, the GMM model has been used. The statistical population of this research is 112 banks from 20 African countries and the financial information of this bank was used during the years 2011–2017. The obtained results show that bank managers have made good use of LLPs for income smoothing and ownership concentration increases income smoothing. Another important result obtained in this research is that in developing countries, foreign investment greatly reduces the powers of bank managers, and with the increase of foreign investment, financial transparency increases.

In Doan et al. [43]‘s attempt to investigate the relationship between government ownership and income smoothing of commercial banks, we see that in developing countries, banks make more efforts to smooth income and the shareholders of these banks are more under the control of the government. Also, in this research, an attempt has been made to examine the relationship between political connections and the effect of government ownership. The results show that profit smoothing in state and non-state banks in developed countries is not much different. To investigate the impact of political goals on income smoothing in state banks, the financial information of election years has been used. The obtained results show that this relationship is meaningful and that this effect exists and works differently in different countries.

Angelucci et al. [8], in a research, seeks to examine the income smoothing of British banks using loan loss provisions. To achieve this goal, the financial information of these banks has been used during the years 1999–2017. The obtained results show that the investigated banks use LLPs for income smoothing. Another important result expressed in this research is that when uncertainty in economic policies is higher, income smoothing will be higher. Also, when economic crises and severe recession occur, income smoothing using LLPs decreases. Of course, it is mentioned in this research that these results were obtained with the knowledge that economic conditions and accounting disclosure rules have a great impact on the level of income smoothing.

Danisman et al. [9] has tried to investigate the relationship between economic policy uncertainty and LLPs. To achieve this goal, GMM and IV models have been used and the statistical population of this research is 6384 US banks. The financial information of these banks has been examined during the years 2009–2019. The obtained results show that when uncertainty increases, LLPs grow in banks. Also, when there is little uncertainty, in the sense that critical conditions have not occurred, income smoothing is done using LLPs. Another important point that is evident in the results is that apart from normal and critical times, there are also uncertain times in which EPU and LLPs have a meaningful and direct relationship and private banks do more income smoothing using LLPs.

Shala et al. [52] tried to investigate the effects of competition on income smoothing in banks for the first time. To achieve this goal, they have used the information of commercial banks in Central and Eastern European countries (CEE) during the years 2004–2015 and the GMM model. The obtained results show that the competition parameter has an inverse relationship with LLP and income smoothing using LLP is not possible in a highly competitive or highly concentrated environment. Also, when there is intense competition, banks try to consider less reserves and their credit risk increases greatly.

Dantas et al. [53] have sought to investigate the effect of government guarantees on the performance of banks in line with income smoothing. To achieve this goal, two parameters of implicit guarantees after the creation of the Eurozone and explicit guarantees granted have been used. The obtained results show that when government guarantees increase, income smoothing in banks decreases and the opposite of this relationship is also true. It is also evident that the risk of banks is greatly reduced by using government guarantees, and in this situation, managers no longer seek income smoothing.

Several studies have highlighted the interaction between political forces and financial reporting in banks. In particular, research such as Doan et al. [43] and Sapienza [40] emphasize that government-owned banks may respond to political objectives—especially during election years—by managing reported earnings to align with policy expectations. These findings support the assumption that political cycles could influence income smoothing behavior, especially when governments act as dominant shareholders. In the context of developing countries, where regulatory institutions may be weaker and political ties more pronounced, such behavior can be even more observable. This study contributes to this literature by investigating whether political incentives, proxied by national election years, amplify income smoothing practices through LLPs, particularly in banks with higher levels of government ownership.

## 3. Materials and methods

**Fig 2:**
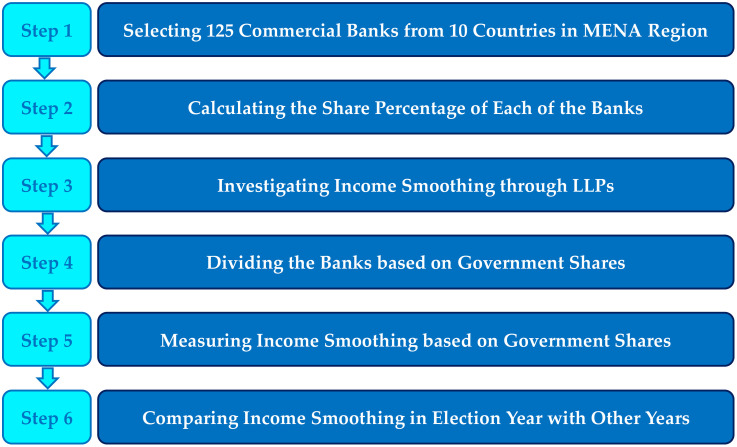
The schematic summary of all steps in the proposed GMM approach.

In this section, to achieve the goal of this research, which is to investigate the influence of political shareholders on the way of allocating resources and exercising power over the management of banks for political and social purposes, an analytical approach using the GMM model is proposed step by step. The financial information of 125 commercial banks from ten countries in the Middle East and North Africa region has been used between 2015 and 2019. To investigate the effect of policy on bank management, by calculating the percentage of government shares in each bank, we examine income smoothing through LLPs using the GMM method. Then, by dividing banks based on the percentage of government shares, we measure income smoothing based on government shares. Finally, to investigate the effect of political factors, we compare income smoothing in the election year with other years. The methodology of the article is shown in Fig 2.

The GMM estimation method is used as one of the most widely used econometric techniques, both in cross-sectional estimation and in panel data estimation, because it is very flexible and requires only weak assumptions. In this model, the intercept of the dependent variable appears as the independent variable on the right side of the equation. In this way, it is possible to re-parameterize the model using the method of dynamic consolidated data and short-term stretching becomes possible.

The estimation technique in the GMM method is an extension of the moment technique that has been extended to other models beyond linear regression. The method of moments is an estimation technique that states that the unknown parameters should be estimated by matching the population moments (which are functions of the unknown parameters) with appropriate sample moments.

The reason for using the GMM method is the advantages of this method compared to other econometric methods, and this method is suitable for at least three reasons. Although the government ownership variable may not vary significantly over time for some banks, it still exhibits variation across banks and across countries, which can be exploited in a panel GMM framework. Moreover, interaction terms with time-varying political variables such as ELECT allow the model to capture dynamic effects even with partially time-invariant covariates. Therefore, while acknowledging potential identification limitations, the use of system-GMM remains justified due to the presence of lagged dependent variables and possible endogeneity in earnings and control variables. Endogenous variables can also be used in this method. One of the ways to control the endogeneity of variables is to use an instrumental variable. An instrument will have the necessary power when it has a high correlation with the investigated variable, while it is not correlated with the error components.

However, finding such a tool is very difficult. One of the advantages of the GMM method is that it allows us to use the intercept of these variables as suitable tools to control endogeneity. The second advantage of this method is that the dynamics in the investigated variable can be included in the model, and the third advantage of this method is that it can be used in time series, cross-sectional and panel data.

To calculate state ownership, we use the method of La Porta et al. [54,55]. In this research, shareholders with more than 10% are considered as influential factors. An important point that can be stated is that only domestic shareholders have been used to examine the smoothing of banks’ income according to the internal policies of each country. The share of government shareholders is measured through the proportion of each shareholder in the percentage owned by the government as follows:


$${SHGOV}_{ij}=\;\sum_{h=\text{1}}^{H}S_{hi}S_{gh}$$

$$S_{hi}S_{gh}\;>\;\text{0}.\text{1}$$

where j = 1...10: is the number of countries in the sample.

h=1…H: indexes banks’ shareholders.

Sh_hi_: is the share of bank i owned by shareholder h.

Sh_gh_: is the share of shareholder h that is owned by a government shareholder.

SHGOV_ij_: The proportion of government shares in the shares of bank i in country j of each year. An important point that should be mentioned here is an example of calculation of government ownership (SHGOV) for two Iranian banks in year t, which is displayed in Table 1.

**Table 1:** Example of the computation of the government ownership (SHGOV) for two banks in Iran in year t.

Bank name	Shareholders	Type	Ownership(%)	Government’s stake in shareholder(%)
EGHTESAD NOVIN BANK PJSC	IRAN CONSTRUCTION INVESTMENT PUBLIC JOINT STOCK COMPANY	Financial company	26.81	60
	BEHSHAHR CAMPANY	Corporate	34.57	74.5
	TEHRAN RENOVATION AND CONSTRUCTION COMPANY	Corporate	24.79	60
	SHERKAT TAMIN MASKAN JAVANAN	Corporate	8.52	41.81
	SAMANEH STRATUS INVESTMENT COMPANY	Financial company	6.57	0
BANK TEJARAT	PROVINCIAL INVESTMENT COMPANIES	Financial company	40	100
	GOVERNMENT OF IRAN	Government	10.14	100
	BFM DEDICATED IRANIAN TRADE MARKETING FUND TRUST	Financial company	14.67	0
	SABA TAMIN INVESTMENT COMPANY	Financial company	14.54	100

The computation of the government ownership variable for Iranian EGHTESAD NOVIN BANK PJSC includes five elements. First, the government of Iran owns a 60% stake in Iran Construction Investment Public Joint Stock Company. This company holds a 26.81% stake in this bank. Second, the government of Iran owns a 74.5% stake in Behshahr Company. Behshahr Company holds a 34.57% stake in this bank. Third, the government of Iran owns a 60% stake in Tehran Renovation and Construction Company. This company holds a 24.79% stake in this bank. Fourth, the government of Iran owns a 41.81% stake in Sherkat Tamin Maskan Javanan. This company holds a 8.52% stake in this bank. Finally, the government of Iran does not have a percentage of shares in the Samaneh Stratus Investment Company.


$${SHGOV}_{EGHTESAD\;NOVIN}=(\text{2}\text{6}.\text{8}\text{1}\%\times \text{0}.\text{6})+(\text{3}\text{4}.\text{5}\text{7}\%\times \text{0}.\text{7}\text{5}\text{4})+(\text{2}\text{4}.\text{7}\text{9}\%\times \text{0}.\text{6})+(\text{8}.\text{5}\text{2}\%\times \text{0}.\text{4}\text{1}\text{8}\text{1})$$

=60.26%

The computation of the government ownership variable for Iranian BANK TEJARAT includes four elements. First, the government of Iran owns a 100% stake in Provincial Investment Companies. This company holds a 40% stake in this bank. Second, government of Iran holds a 100% stake in this bank. Third, the government of Iran does not have a percentage of shares in the Bfm Dedicated Iranian Trade Marketing Fund Trust. Finally, the government of Iran owns a 100% stake in Saba Tamin Investment Company. This company holds a 14.54% stake in this bank


$${SHGOV}_{BANK\;TEJARAT}=(\text{4}\text{0}\%\times \text{1})+(\text{1}\text{0}.\text{1}\text{4}\%\times \text{1})+(\text{1}\text{4}.\text{5}\text{4}\%\times \text{1})=\text{6}\text{4}.\text{6}\text{8}\%$$

To classify government ownership in different banks, two methods of simple classification and clustering have been used, but before that, we calculate the share of government shareholders in the investigated banks at the end of each year. To check the amount of government shares and determine the majority of shares (more than 50%, which takes the value of one), we use the threshold criterion.

Consistent with Bouvatier et al. [36], we use the hierarchical clustering (HAC) method to more precisely classify banks in terms of the degree of government ownership concentration. The hierarchical clustering method is based on an aggregation algorithm, which allows merging clusters according to the Euclidean distance similarity of multiple evaluated dimensions. In this case, we focus on clustering based on the ratio of government shares. To determine the distance between clusters consisting of several banks, we use the single link rule, in which banks may be transferred from one cluster to another with the passage of time and the difference in the percentage of government shares.

Based on the clustering method of Husson & Josse [56], we consider three separate clusters. Banks in the first cluster have a small proportion of state owners who do not hold the majority of controlling shares. Banks in the second cluster have a higher proportion of government shares (on average) and banks in the third cluster have a very high degree of government ownership. Finally, we are faced with three relative variables GOC_k_ (k = 1, 2, 3) in the specifications of the main model, and the variables GOC_1_, GOC_2_, and GOC_3_ are virtual variables that show the proportions of government ownership.

Before focusing on the government’s role in controlling bank resources, we need to estimate the basic relationship between bad debt provision and bank profit for the period 2015–2019. This method is similar to those of Fonseca & Gonzalez [50], Bikker & Metzemakers [57] and Kanagaretnam et al. [58]. To separate the discretionary and non-discretionary components of the LLPs, we use the method of Beaver & Engel [19], Ahmed et al. [59] and Anandarajan et al. [60].

Capital management and income smoothing are optional components that are mostly used to manage banks, but on the other hand, non-optional components are used to cover credit losses. We consider the following model:


$${LLP}_{i.j.t}=\beta_{\text{0}}+\beta_{\text{1}}{LLP}_{i.j.t-\text{1}}+\beta_{\text{2}}{EARN}_{i.j.t}{+\alpha }^{\prime }{BankControls}_{i.j.t}$$

$$+\;\gamma^{\prime }{BankRegulationControl}_{j.t}+\rho^{\prime }{MacroControls}_{j.t}+e_{i.j.t}$$ (1)

When the income of a bank is low, the LLP parameter can be used to manipulate the income, and according to this issue, the EARN_i,j,t_ parameter should be examined. The LLP parameter is an optional component and we expect that after income smoothing using LLP, the coefficient of EARN ($\beta_{\text{2}}$) will be positive and significant.

For the non-discretionary component of LLPs, according to the method of Bouvatier et al. [37], the ratio of loans to total assets (L_i,j,t_) and loan growth rate (ΔL_i,j,t_) as a control variable to determine bank credit risk We use, this variable should be positively related to LLPs when banks make general LLPs from their loan expansions.

Stiroh & Rumble [61] and Micco et al. [46] show that the share of deposits and non-interest income is closely related to bank performance, but bank size and lagged ratio of equity variables cause financial problems and the probability of bank bankruptcy, respectively. If banks use LLPs to manage the capital requirement in Basel 1 (and even in Basel 2), We expect the coefficients of BANKSIZE and EQUITY to be negative.

Fonseca & Gonzalez [50] and Kanagaretnam et al. [58] show that one of the reasons for the transparency of income is official supervision. The weaker the accounting system and official supervision, the greater the possibility of income manipulation. One of the possible reasons is that financial statements with fewer details create an opportunity for income smoothing for banks. Considering this factor, we use other indicators in our model. CAPSTR_j,t_, ACCINF_j,t,_ and SUPPOWj,_t_ were taken from the corresponding World Data Bank [62,63]. The World Bank has designed questions to find out the regulatory requirements and regulations and categorized the level of supervision of the banking system of different countries based on the answers to these questions.

CAPSTR_j,t_: It measures the level of regulatory requirements related to banks’ capital, shows some elements of risk, and subtracts some market value losses from the determinants of capital adequacy.

ACCINF_j,t_: Bank accounting informative on the financial statement contains audited or unpaid interest or principal on non-performing loans and measures the condition details of the consolidated income statement.

SUPPOWj,_t_: Measures the authority of bank supervisors to take specific actions against bank management to prevent and correct problems. Higher values of these indicators indicate more bank regulation and formal supervision.

Since the high level of supervision increases the quality of banks’ reporting, we expect the coefficients of these variables to be negative. And we use macroeconomic controls in the model specification to control the effects of economic growth and inflation rate on the macroeconomic level.

To estimate the income smoothing hypothesis, we use the GMM estimator according to the specification of the dynamic panel model [64]. This system GMM estimator was introduced by Holtz-Eakin et al. [65] and Arellano & Bond [66] developed in a series of papers by Arellano & Bover [67] and Blundell & Bonds [68], we perform the dynamic GMM regression using the forward orthogonal deviations transformation, which allows us to compute the estimator for all observations of a variable.

In addition to the estimation procedure, we conduct standard diagnostic tests to ensure the reliability of our GMM estimates. Specifically, we report the Hansen test of overidentifying restrictions to verify the validity of instruments, and the Arellano-Bond test for second-order autocorrelation (AR(2)) in the residuals. The results of these tests, presented in the robustness section, support the consistency and appropriateness of our instrument set.

For income smoothing, we analyze a baseline model with the aim of comparing the effect of state ownership structure and then we examine the effects of elections in years when elections are held and in years when they are not.

According to Equation (1) and according to the difference between state ownership in simple classification and clustering methods, the models are analyzed with the aim of investigating the impact of state shares on bank management that we should use bank data and standard bank performance indicators to rewrite the following equation:


$${LLP}_{i.j.t}=\beta_{\text{0}}+\beta_{\text{1}}{LLP}_{i.j.t-\text{1}}+\beta_{\text{2}}{EARN}_{i.j.t}+\sum_{k=\text{1}}^{\text{2}}\beta_{k+\text{2}}{EARN}_{i.j.t}\times {GO}_{k.i.j.t}+\sum_{k=\text{1}}^{\text{2}}\beta_{k+\text{4}}{GO}_{k.i.j.t}$$

$$+\;\alpha^{\prime }{BankControls}_{i.j.t}+\gamma^{\prime }\;{BankRegulationControl}_{j.t}+\rho^{\prime }\;{MacroControls}_{j.t}+e_{i.j.t}$$ (2)

$${LLP}_{i.j.t}=\beta_{\text{0}}+\beta_{\text{1}}{LLP}_{i.j.t-\text{1}}+\beta_{\text{2}}{EARN}_{i.j.t}+\sum_{k=\text{1}}^{\text{3}}\beta_{k+\text{2}}{EARN}_{i.j.t}\times {GOC}_{k.i.j.t}+\sum_{k=\text{1}}^{\text{3}}\beta_{k+\text{5}}{GOC}_{k.i.j.t}$$

$$+\;\alpha^{\prime }{BankControls}_{i.j.t}+\gamma^{\prime }\;{BankRegulationControl}_{j.t}+\rho^{\prime }\;{Macro\;Controls}_{j.t}+\;e_{i.j.t}$$ (3)

Multiplying the two parameters EARN and GO indicates the difference in income smoothing between banks with a majority of government and non-government shares. In order to determine the state ownership of a bank, cluster dummy variables are used in the Equation (3) model, where cluster 1 (with low ownership share) is defined as the reference category. Considering the transparency of state banks, in this section, we seek to investigate the impact of additional factors on the relationship between income smoothing behavior and bank ownership.

Some parts of the existing literature suggest that banks and firms with government shareholders are less stable and possibly more vulnerable to elections. We include an election year variable (ELECT) as a measure of political participation in the equation, with information from the World Bank’s database of political institutions based on Micco et al. [46] and Julio & Yook [51]. It is a database of annual legislative information and other comparative indicators of election results, legislative, executive, and political ideology. If the necessary information is not available in the annual database of the World Bank, we supplement the information from various Internet sources. While primary election data were sourced from the World Bank Database of Political Institutions, for countries where gaps existed, we supplemented this information by cross-validating multiple credible sources such as national election commission websites, government portals, and reputable international news agencies. A detailed list of sources used for each country and year is provided in the supplementary appendix to enhance transparency and replicability.

Model (4) and model (5) which is as follows:


$${LLP}_{i.j.t}=\beta_{\text{0}}+\beta_{\text{1}}{LLP}_{i.j.t-\text{1}}+\beta_{\text{2}}{EARN}_{i.j.t}+\sum_{k=\text{1}}^{\text{2}}\beta_{k+\text{2}}{EARN}_{i.j.t}\times {GO}_{k.i.j.t}$$

$$+\;\sum_{k=\text{1}}^{\text{2}}\beta_{k+\text{4}}{{EARN}_{i.j.t}\times GO}_{k.i.j.t}\times {ELECT}_{j.t}+\;\sum_{k=\text{1}}^{\text{2}}\beta_{k+\text{6}}{GO}_{k.i.j.t}+\beta_{\text{9}}{ELECT}_{j.t}$$

$$+\;\sum_{k=\text{1}}^{\text{2}}\beta_{k+\text{9}}{GO}_{k.i.j.t}\times {ELECT}_{j.t}+\alpha^{\prime }{BankControls}_{i,j,t}+\gamma^{\prime }{BankRegulationControl}_{j.t}$$

$$\rho^{\prime }\;{MacroControls}_{j.t}+e_{i.j.t}$$ (4)

$$+\;{LLP}_{i.j.t}=\beta_{\text{0}}+\beta_{\text{1}}{LLP}_{i.j.t-\text{1}}+\beta_{\text{2}}{EARN}_{i.j.t}+\sum_{k=\text{1}}^{\text{3}}\beta_{k+\text{2}}{EARN}_{i.j.t}\times {GOC}_{k.i.j.t}\;$$

$$+\;\sum_{k=\text{1}}^{\text{3}}\beta_{k+\text{5}}{{EARN}_{i.j.t}\times GOC}_{k.i.j.t}\times {ELECT}_{j.t}+\sum_{k=\text{1}}^{\text{3}}\beta_{k+\text{8}}{GOC}_{k.i.j.t}+\beta_{\text{1}\text{2}}{ELECT}_{j.t}\;$$

$$+\;\sum_{k=\text{1}}^{\text{3}}\beta_{k+\text{1}\text{2}}{GOC}_{k.i.j.t}\times {ELECT}_{j.t}+\alpha^{\prime }{BankControls}_{i.j.t}+\gamma^{\prime }{BankRegulationControl}_{j.t}\;$$

$$\rho^{\prime }{MacroControls}_{j.t}+e_{i.j.t}$$ (5)

ELECT is a virtual variable, it takes the value of one in the election year in country j and zeroes otherwise. In countries with a presidential system, the president usually has the highest executive power. In countries with a parliamentary system, the executive power is in the hands of cabinets assigned to the parliament. Therefore, in this research, in the absence of presidential elections, we use parliamentary elections. According to the theory of income smoothing due to political goals and the difference in banks’ performance, we expect the coefficients of EARN × GO × ELECT and EARN × GOC × ELECT to be significant and positive [41].

To ensure the results of the models, we examine income smoothing in the years before and after the elections. Banks with government shareholders may use their resources to obtain political support for electoral purposes, so the income smoothing behavior for these banks should be changed before the elections so that they can obtain the necessary banking resources for these purposes [69,70].

We previously used the ELECT dummy variable in the models to examine pre- and post-election years, we use the dummy variable ELECT(−1) means one year before the election, and the dummy variable ELECT(+1) means one year after the election, and the dummy variable ELECT(+2) means two years after the election.

Based on the results obtained from the research of Micco et al. [46], we expect the income smoothing behavior for state-owned banks to start in the year before the election and increase much more in the election years and decrease in the second year after the election. Julio & Yook [51] shows that due to political uncertainty, companies face lower investment rates in election years and refrain from investing until political stability.

## 4. Empirical application

The impact of government policy on income smoothing has been analyzed using descriptive statistics of independent and control variables and economic information during the years 2015–2020. LLP, EARN and ΔL variables are the ratio of LLPs to total assets, the ratio of pre-tax profit to total assets and loan growth rate, respectively. Also, variable L shows the ratio of loans to total assets. It should also be noted that GO is a dummy variable. DDEP, NONINT, EQUITY, REALGDP and INFLATION variables are respectively demand deposits, ratio of non-interest income to total assets, ratio of equity to total assets, gross domestic product and inflation with the base year of 2010.

Table 2 provides summary statistics regarding LLPs, government ownership, and other control variables. The mean value of our LLPs is 0.023 percent, whereas EARN is 3.742 percent. The mean value of GO is 33.978 percent, on average, L is 0.0843 percent whereas ΔL is 51.708 percent. The mean of bank regulation control Including CAPSTR, ACCINF, and SUPPOW are 3.000, 4.005, and 8.677, respectively. On average, Macro Controls variables include REALGDP and INFLATION are 7645.864 and 4.662 percent, respectively.

**Table 2:** Descriptive statistics of independent and control variables.

Variables	Mean	SD	Maximum	Minimum
LLP_i,j,t-1_	0.023	0.004	2.538	−0.027
EARN	3.742	0.032	1583.838	−0.276
GO	33.978	22.290	100.000	0.000
L	0.0843	0.059	5.455	−0.914
ΔL	51.708	56.560	93.380	0.010
NONINT	−5763.842	0.013	696139.6	−2882609
DDEP	0.492	0.180	20.178	−0.368
BANKSIZE	12554660	5144126	1.07E + 08	162617
EQUITY	12.508	9.980	93.200	−196.165
CAPSTR	3.000	3.001	6.000	0.000
ACCINF	4.005	6.000	6.000	0.029
SUPPOW	8.677	13.000	13.000	0.032
REALGDP	7645.864	4426.214	29869.55	0.066
INFLATION	4.662	3.271	12.484	−0.876

We have calculated the percentage of government shares of each bank in each country using the method of La Porta et al. [54,55]. Table 3 shows the average percentage of government shares in ten countries of MENA.

**Table 3:** Average of SHGOV for countries.

Countries	Mean of SHGOV	No. banks
Algeria	74.68	10
Bahrain	49.46	10
Egypt	43.17	12
Iran	46.50	22
Iraq	9.98	13
Jordan	16.65	11
Kuwait	26.93	6
Lebanon	16.84	21
Morocco	35.50	11
Tunisia	28.98	12

Among the studied countries, Algeria has the highest percentage of government shares with an average of 74.68% and Iraq has the lowest percentage of government shares with an average of 9.98%. It should also be noted that in this study, banks with more foreign shareholders and under the supervision of foreign countries were excluded from the study to prevent foreign political interference with the political effect of the elections in each country.

Based on model (1), we examined income smoothing through LLPs regardless of the percentage of government shares in all commercial banks of ten countries in the MENA. Table 4 results from the model (1), which is a study of income smoothing.

**Table 4:** Estimation of the model (1).

	Variables	Coefficient
Independent Variable	LLP_i,j,t-1_	10.525
	EARN	0.174
Bank Controls	L	0.230
	ΔL	−0.232
	NONINT	1.396
	DDEP	−0.025
	BANKSIZE	−0.013
	EQUITY	−0.069
Bank Regulation Controls	CAPSTR	0.121
	ACCINF	0.080
	SUPPOW	−0.001
Macro Controls	REALGDP	0.230
	INFLATION	−0.232

Therefore, based on the overall significance of the model, it can be said that there is a fundamental relationship between LLPs and income smoothing.

LLP_i,j,t-1_ has a positive coefficient, so it can be said that LLP_i,j,t-1_ has a positive and significant effect on the dependent variable. The coefficient of EARN is positive, so it can be said that EARN has a positive and significant effect on the dependent variable. Positive LLP_i,j,t-1,_ and EARN indicate income smoothing.

Among the bank control variables L and NONINT, they have a positive and significant effect on the dependent variable, but DDEP, BANKSIZE, ΔL and EQUITY have a negative effect on the dependent variable.

According to these results, it can be said that income smoothing is done more in small banks and the income from lending has had a positive effect on the dependent variable. Also, these results are consistent with the results of previous research [69,70].

Among Bank Regulation Control variables, CAPSTR and ACCINF, have a positive effect on the dependent variable, while SUPPOW has a negative effect, and it states that external supervision of banks’ performance will prevent income smoothing, positivity of the other two regulatory components indicates the need for better implementation of supervisory laws.

We estimated model (1) for commercial banks in Iran. Table 5 shows the results of income smoothing in commercial banks of Iran.

**Table 5:** Estimation of the model (1) for Iranian banks.

	Variables	Coefficient
Independent Variable	LLP_i,j,t-1_	0.136
	EARN	0.036
Bank Controls	L	0.045
	ΔL	0.051
	NONINT	0.432
	DDEP	−0.039
	BANKSIZE	−0.0001
	EQUITY	0.137
Bank Regulation Controls	CAPSTR	0.058
	ACCINF	−0.106
	SUPPOW	−0.002
Macro Controls	REALGDP	0.009
	INFLATION	−0.034

Considering that the LLP_i,j,t-1_ coefficient is 0.13, it can be said that this variable has a positive and significant effect on the dependent variable.

The EARN coefficient is 0.03, so it can be said that the independent variable has a positive and significant effect on the dependent variable.

The positive coefficients of LLP_i,j,t-1,_ and EARN indicate that income smoothing had been done in Iranian banks.

The L coefficient is equal to 0.04, so it can be said that L has a positive and significant effect on the dependent variable.

The positive coefficient of ΔL shows that this variable has a positive and significant effect on the dependent variable.

The negative coefficient of DDEP indicates the negative impact of this variable on the dependent variable.

According to the general estimation of the model (1) and the alignment of the results with the estimation of the model (1) for Iran, it can be said that operations related to loans and non-interest incomes have a positive effect on income smoothing, as well as the regulatory components of ACCINF and SUPPOW in Iran have prevented income smoothing and with the increase in bank assets Income smoothing operations are reduced.

Based on the model (2), we divided the banks into two groups according to the percentage of government shares, the percentage of government shares is higher than 50% and the percentage of government shares is less than 50%, to examine the effect of the maximum ownership of government shares on income smoothing. Table 6 shows the impact of state ownership on income smoothing based on simple clustering in ten countries in the MENA.

**Table 6:** Estimation of the model (2).

	Variables	GO > 50%	GO < 50%
Independent Variable	LLP_i,j,t-1_	−16.923	0.039
	EARN	1.157	0.036
	EARN × GO	10.221	0.365
	GO	0.813	−0.434
Bank Controls	L	−0.0006	−0.038
	ΔL	−3.921	−0.004
	NONINT	12.605	−0.0003
	DDEP	37.030	0.158
	BANKSIZE	−0.357	0.083
	EQUITY	−8.286	−0.096
Bank Regulation Controls	CAPSTR	31.895	−0.0003
	ACCINF	0.664	0.231
	SUPPOW	1.821	0.287
Macro Controls	REALGDP	−0.017	−0.014
	INFLATION	0.019	0.054

After estimating model (1) and checking income smoothing, in the model (2) the percentage of government shares is divided into two groups and the two variables GO and EARN **×**GO are added to the independent variables of the research.

In general, according to the estimation results of the model, the coefficient of LLP_i,j,t-1_, is positive in banks with more than 50% government shares And the coefficient of the EARN variable is positive in this type of banks and it can be said that the managers of government-controlled banks use LLPs to manage the banks’ income to banks to achieve more private interests.

Also, EARN in banks with less than 50% government shares has a positive coefficient. It can be said that managers of banks with less than 50% government ownership, like banks with more than 50% government ownership, use LLPs to manage the banks’ income to achieve more private interests.

The coefficient of EARN **×**GO was positive in two groups, but this coefficient was very high in banks with more than 50% government shares, which indicates higher income smoothing in these banks.

Contrary to the results of model (1), in this model, according to the significant coefficient of the BANKSIZE report, the increase in bank assets has a positive effect on LLPs in banks with government shares less than 50%. But this ratio is negative in banks with government shares above 50% and it is consistent with the results of model (1).

Also, EQUITY is negative in both groups, which indicates a negative impact on the dependent variable.

Considering that Bank Regulation Control is generally defined by the government and politicians to monitor the implementation of laws, the lack of attention to regulatory measures, the positive coefficient of these variables is acceptable.

REALGDP has a negative coefficient in both groups, which indicates the lack of impact of banks on development, and INFLATION is positive in banks with government shares of more than 50%, which can indicate an increase in inflation through the activity of these banks.

In order to more closely examine the effect of government ownership on income smoothing, we use model (3) that divides the percentage of government shares based on hierarchical agglomerative clustering (HAC).

Table 7 examines the impact of the percentage of government shares based on hierarchical agglomerative clustering (HAC) on income smoothing in ten countries in MENA.

**Table 7:** Estimation of the model (3).

	Variables	GOC 3	GOC 2	GOC 1
Independent Variable	LLP_i,j,t-1_	0.317	0.036	−0.146
	EARN	4.203	0.036	0.075
	EARN × GOC	2.646	0.005	0.004
	GOC	0.036	37.030	0.158
Bank Controls	L	0.001	−0.106	−0.028
	ΔL	0.195	−0.002	0.009
	NONINT	1.23E10	0.009	−0.008
	DDEP	−0.075	−0.034	−0.035
	BANKSIZE	17.402	0.069	0.013
	EQUITY	−1.464	0.019	−0.0001
Bank Regulation Controls	CAPSTR	11.161	0.005	0.171
	ACCINF	−0.021	0.049	−0.047
	SUPPOW	−0.004	−0.453	−0.026
Macro Controls	REALGDP	−0.083	−0.035	0.231
	INFLATION	0.620	0.0002	0.075

The LLP_i,j,t-1_ was positive in two groups, 3 and 2, so it can be said that this ratio has a positive effect on the dependent variable.

EARN coefficient is positive in three groups and has a direct impact on the dependent variable and shows that income smoothing has been done

In the third group, the coefficient of EARN × GOC is positive. Therefore, it can be said that there is a high level of income smoothing through LLPs in this group. Regarding the second group, its EARN × GOC coefficient is 0.036. In the first group, the EARN × GOC coefficient is 0.075. Therefore, the highest level of income smoothing through LLPs occurs in the third group.

The positive coefficients of L and ΔL indicate the positive effect of lending on the dependent variable. Considering that these coefficients are negative in group 2, the results of model (2) can also be accepted, because part of the banks in group 2 in the model (2) is included in the group of banks with more than 50% government shares.

The BANKSIZE coefficient is positive in three groups, which indicates the increase in income smoothing with the increase in bank assets. This ratio was much higher in group 3, which indicates a greater effect.

CAPSTR has a positive coefficient, which indicates the lack of proper attention to capital regulatory laws. ACCINF and SUPPOW have negative coefficients, although these coefficients are small, they indicate better supervision in these areas.

REALGDP has a negative coefficient in groups 3 and 2, which indicates that the banks of these groups did not have a positive impact on development, but in group 1, this coefficient is positive and indicates a positive impact on development. The INFLATION coefficient is positive in all three groups, showing the effect of the increase in inflation.

In the next step, we use the election factor to examine the effect of policy on income smoothing. In model (4), the election factor is tested based on simple clustering. Table 8 shows the impact of election on income smoothing in banks the percentage of government shares is more than 50 percent and the percentage of government shares is less than 50 percent in ten countries of MENA.

**Table 8:** Estimation of the model (4).

	Variables	GO > 50%	GO < 50%
Independent Variable	LLP_i,j,t-1_	0.317	0.036
	EARN	4.203	0.036
	EARN × GO	2.646	0.005
	GO	−12.833	−0.453
	ELECT	0.018	0.432
	GO× ELECT	0.511	0.039
	EARN × GO × ELECT	0.827	−0.0001
Bank Controls	L	0.001	−0.106
	ΔL	0.195	−0.002
	NONINT	1.23E10	0.009
	DDEP	−0.075	−0.034
	BANKSIZE	17.402	0.069
	EQUITY	−1.494	0.019
Bank Regulation Controls	CAPSTR	11.161	0.005
	ACCINF	−0.021	0.049
	SUPPOW	−0.004	−0.453
Macro Controls	REALGDP	−0.083	−0.035
	INFLATION	0.620	0.0002

LLP_i,j,t-1_ has a positive coefficient in both groups, so it can be said that this ratio has a positive effect on the dependent variable.

Also, EARN is positive in both groups, which indicates income smoothing, and this coefficient is much higher in the group with the percentage of government shares above 50%, which shows that more income smoothing has been done in this group.

The coefficient of the two variables EARN × GO × ELECT and EARN × GO, which we have used as variables to identify income smoothing, is positive in banks with more than 50% government shares. And it shows that income smoothing has been done in these banks in the election years.

Loan ratios including L and ΔL in banks with more than 50% government shares have a positive coefficient, which indicates the direct effect of lending operations on the dependent variable.

BANKSIZE with a positive coefficient expresses the effect of increasing bank assets on increasing income smoothing. Also, this coefficient has been higher in banks where government shares are above 50%, which shows the greater effect of increasing bank assets on income smoothing.

ACCINF and SUPPOW have a negative coefficient and it shows that the implementation of these supervisions has reduced income smoothing, but CAPSTR has a positive coefficient, which indicates the need for stricter implementation of capital regulations.

The Macro Control variables also confirmed the findings of the previous models because the coefficient of REALGDP was negative in both groups and indicated the lack of impact of banks on the development of the country during the election year. The INFLATION coefficient is positive, which indicates the impact of banks on the increase in inflation.

Based on the results of this model, the effect of the political factor on income smoothing has been confirmed. And our robustness test revealed the fact that income smoothing for banks with government-controlled shareholders can be affected by the potential instability of political institutions.

In model (5), using HAC, we divide banks into three groups based on the percentage of government shares and examine the effect of the election factor on these three groups. Table 9 shows the impact of the election on income smoothing in banks of ten countries in MENA.

**Table 9:** Estimation of the model (5).

	Variables	GOC 3	GOC 2	GOC 1
Independent Variable	LLP_i,j,t-1_	0.073	0.129	−0.034
	EARN	0.399	0.022	0.064
	EARN × GOC	0.231	−0.004	4.90E-05
	GOC	−0.0004	0.029	−0.030
	ELECT	−0.111	−0.0001	0.601
	GOC× ELECT	−0.028	−0.009	−0.590
	EARN × GOC× ELECT	0.959	−0.098	0.299
Bank Controls	L	0.019	−0.0004	0.283
	ΔL	0.006	−0.115	−0.012
	NONINT	0.048	0.016	−0.071
	DDEP	0.446	−0.125	−0.0006
	BANKSIZE	0.036	0.274	−0.106
	EQUITY	−0.0004	−0.036	−0.129
Bank Regulation Controls	CAPSTR	0.312	0.045	−0.068
	ACCINF	−0.068	−0.024	−0.003
	SUPPOW	0.004	0.711	−0.143
Macro Controls	REALGDP	−0.029	−0.097	0.149
	INFLATION	−0.017	−0.097	−0.007

LLP_i,j,t-1_ has a positive coefficient in two groups of 3 and 2 and indicates the positive effect on the dependent variable.

The coefficient of the EARN is positive in three groups and confirms the income smoothing theory as in the previous research models [41] and this coefficient is bigger in group 3, which has the most government shareholders.

The coefficient of the two variables EARN × GOC and EARN × GOC × ELECT, which shows the smoothing of income in banks with more government shares and the effect of the election year, has been positive in three groups, and this coefficient is in group 3, which includes banks with the most government shares, is more than the other two groups.

Loan-related variables including L and ΔL have a positive coefficient in group 3 and show the direct effect of these two ratios on the dependent variable.

The positive coefficient of BANKSIZE also refers to the increase in income smoothing in banks with more assets.

Two variables CAPSTR and SUPPOW both have positive coefficients in two groups of 3 and 2, which show that in the election year, the implementation of laws and monitoring of banking activities was not done properly. But the negativity of the CAPSTR coefficients for group 1 refers to the implementation of laws in banks with the lowest percentage of government shares, and it is another aspect of the lack of proper implementation of supervisory operations in banks with the highest percentage of government shares. However, ACCINF has a negative coefficient for three groups, and the implementation of this monitoring reduces income smoothing.

The macro control variable, REALGDP coefficient is negative for groups 3 and 2 and indicates the lack of influence of banks with a higher percentage of government shares on the development of the country in the election year. While this ratio was positive in the election year in group 1, which has the lowest percentage of government shares, it shows the positive impact of these banks on the development of the country.

The INFLATION was negative in three groups, which indicates the lack of impact of these banks on the increase in inflation in the election year.

It should be noted that the values of the coefficients of two variables EARN and EARN × GOC × ELECT have increased significantly in two groups 1 and 2 compared to one year before the election which is analyzed below.

To ensure the results of the policy’s impact on smoothing the income of the model (5), we examine one year before the election, one year after the election and two years after the election.

Table 10 shows the results of the analysis of the model (5) for one year before the elections in ten countries of MENA.

**Table 10:** Estimation of the model (5) one year before the election.

	Variables	GOC 3	GOC 2	GOC 1
Independent Variable	LLP_i,j,t-1_	18.000	0.036	0.036
	EARN	25.256	−0.005	−0.005
	EARN × GOC	−12.833	−0.038	−0.038
	GOC	134.827	0.050	0.050
	ELECT(−1)	0.490	−0.0003	−0.0003
	GOC× ELECT(−1)	−32.815	0.158	0.158
	EARN × GOC × ELECT(−1)	−0.712	0.083	0.083
Bank Controls	L	1.265	−0.096	−0.096
	ΔL	15.754	−0.0003	−0.0003
	NONINT	−8.286	0.231	0.231
	DDEP	31.895	0.036	0.075
	BANKSIZE	0.664	0.005	0.004
	EQUITY	1.821	0.051	0.073
Bank Regulation Controls	CAPSTR	−0.017	0.432	0.399
	ACCINF	0.664	0.039	0.035
	SUPPOW	1.821	−0.0003	−0.0004
Macro Controls	REALGDP	−0.017	0.158	0.231
	INFLATION	1.436	−0.586	0.658

The LLP_i,j,t-1_ has the highest coefficient in group 3, which includes banks with the most government shares. This ratio is also positive in the other two groups. And it shows the direct effect of this ratio on the dependent variable.

The EARN in the third group, which has the most government shares, is 25.25, which shows the high impact of this factor on the dependent variable. The coefficient of this ratio was also positive in the other two groups. And it shows the positive effect of this ratio on the dependent variable, the positivity of the above two ratios indicates income smoothing.

The EARN × GOC has a negative coefficient in three banking groups, which indicates the lack of influence of government ownership in smoothing income in the year before the elections.

Also, the EARN × GOC × ELECT(−1) in group 3 has a coefficient of −0.712, which, unlike the other two groups that have a positive coefficient, this factor has a negative effect on the dependent variable in group 3.

Ratios related to lending operations in group 3 have a positive coefficient and indicate the positive effect of these two ratios on the dependent variable. However, these ratios are negative in groups 1 and 2 and indicate the negative impact of lending on the dependent variable.

The coefficient of the BANKSIZE in three banking groups is positive and it shows that the increase of bank assets in the three groups will increase income smoothing.

Among the Bank Regulation Control variables, the CAPSTR in group 3 has a negative coefficient, which indicates the implementation of the supervisory rules on bank capital in this group. In groups 2 and 1, the SUPPOW is negative, which indicates more supervisory power over these banks. The coefficient of ACCINF was positive in three groups, which indicates the need for better implementation of accounting supervision rules.

The coefficient of REALGDP in group 3 is negative, which shows that the banks with the most government shares do not have a positive effect on the development of the country. But this coefficient is positive for groups 2 and 1 and it shows the positive impact of these banks on the development of the country.

The coefficient of the INFLATION is positive in all three groups and indicates the increase of inflation in line with the banking activity of the countries.

As a result, income smoothing in the year before the election occurs in banks with the most government shareholders more than other groups and other years, and it shows that the managers of these banks have done income smoothing since the year before the election for political gain. It can be concluded banks that are controlled by the government are not very stable and undergo many changes based on different political goals.

Table 11 shows the results of the analysis of the model (5) for one year after the election in ten countries of MENA.

**Table 11:** Estimation of the model (5) for one year after the election.

	Variables	GOC 3	GOC 2	GOC 1
Independent Variable	LLP_i,j,t-1_	0.813	0.961	−0.566
	EARN	−0.0006	0.435	0.061
	EARN × GOC	−0.357	0.039	0.066
	GOC	12.601	0.301	−0.256
	ELECT(+1)	0.664	0.004	−0.014
	GOC × ELECT(+1)	1.821	0.069	0.029
	EARN × GOC × ELECT(+1)	−0.017	0.037	−0.009
Bank Controls	L	1.265	0.083	−0.022
	ΔL	15.754	−0.165	−8.18E-05
	NONINT	−8.286	−1.478	−0.005
	DDEP	31.895	0.197	−0.0005
	BANKSIZE	0.664	0.004	31.895
	EQUITY	1.821	0.312	0.664
Bank Regulation Controls	CAPSTR	−0.017	0.197	1.821
	ACCINF	0.664	0.004	−0.017
	SUPPOW	1.821	0.069	1.265
Macro Controls	REALGDP	−0.017	0.895	−8.286
	INFLATION	1.286	0.026	1.046

LLP_i,j,t-1_ had a positive coefficient in two groups, 3 and 2, which indicates the positive effect of this ratio on the dependent variable. In group 1, this ratio has a negative coefficient, considering that it is the year after the elections and these banks have done more income smoothing in the previous year, it can be said that this year they are looking to eliminate the effects of the previous year’s smoothing.

The EARN coefficient in group 3 is equal to −0.0006, which indicates the negative impact and reduction of income smoothing in this group one year after the election. However, in the other two groups, this coefficient is still positive and indicates its positive effect on the dependent variable.

The coefficients of EARN × GOC and EARN× GOC × ELECT(+1) in group 3 are negative and it can be said that banks are trying to eliminate the effects of elections. The first ratio in two groups, 2 and 1, has a positive coefficient, which means that government stocks still have a positive effect on income smoothing. However, the second ratio in group 2 has a positive coefficient, which indicates the impact of the elections even in the following year in this group, and the coefficient of the second ratio for group 1 is negative but very small.

The CAPSTR coefficient is negative only in group 3 and it shows the better implementation of capital monitoring rules of this group of banks. The coefficient of ACCINF was negative only in group 1, which includes banks with the lowest state shares, and it indicates a better implementation of regulatory laws in this group of banks. The coefficient of SUPPOW was positive in all three groups, which can be said that due to the elections and the difference in the implementation of laws, the power of official supervision over the banks’ activities has decreased.

The REALGDP has a positive coefficient only in group 2. The positive coefficient of this ratio shows the positive impact of banks on the development of the country. The INFLATION has a positive coefficient in all banking groups and it shows the effect of banking activity on the increase in the inflation rate.

It should be noted that the political outcome of the election had an impact even in the year after the election. Proper management of bank resources due to the spread of political events and their successive effects will make it difficult for banks to properly allocate resources.

Table 12 shows the results of the analysis of the model (5) for two years after the election in ten countries of MENA.

**Table 12:** Estimation of the model (5) for two years after the election.

	Variables	GOC 3	GOC 2	GOC 1
Independent Variable	LLP_i,j,t-1_	2.295	0.019	0.019
	EARN	2.646	0.005	0.006
	EARN × GOC	0.827	0.0002	−0.0004
	GOC	0.619	0.049	0.048
	ELECT(+2)	0.001	0.171	−0.068
	GOC × ELECT(+2)	0.195	−0.047	0.004
	EARN× GOC × ELECT(+2)	1.23E10	−0.026	−0.029
Bank Controls\	L	−0.075	0.231	0.287
	ΔL	17.402	0.235	−0.014
	NONINT	−1.494	0.026	0.027
	DDEP	11.161	−0.014	−0.022
	BANKSIZE	−0.021	0.029	−0.036
	EQUITY	−0.004	−0.009	−0.0004
Bank Regulation Controls	CAPSTR	−0.083	−0.022	0.312
	ACCINF	0.620	−8.18E-05	0.021
	SUPPOW	1.821	−0.005	−0.0005
Macro Controls	REALGDP	17.402	0.0002	−0.0004
	INFLATION	−0.017	−0.0005	0.710

In the two years after the election, the coefficients are similar to the one year before the election, and group 3 has the largest positive coefficient related to LLPs and EARN. These results show that more income smoothing has been done in banks with the most government shares.

EARN × GOC was positive in two groups 3 and 2, which indicates the positive effect of more government ownership on income smoothing. This coefficient was negative in group 1, which includes banks with the lowest percentage of government shares, although this coefficient was small.

EARN × GOC × ELECT(+2) is positive only in group 3 and shows the influence of the political factor, and this ratio is negative in groups 2 and 1, which indicates removing the effects of elections in this group of banks.

By re-estimating model (5) of the research, we observed that in the year before the election, income smoothing was less than in the years after the election, but in group 3, which includes banks with the most government shares, the two coefficients of LLPs and EARN, which are factors for identifying income smoothing in the previous year of election It has been very big, which shows that these banks have been affected by political factors since the year before the elections.

the highest leveling of income in the year before the election happened in the banks that have the most state shares. In the year of the election, the highest leveling of income smoothing happened in two groups 2 and 1, and the political effects in the year of the election were transferred to one year after the election. And in the year after the election, compared to the year before the election, we faced more income smoothing, and these effects decreased in the two years after the election.

**Theorem 1.** *Most of the shares of banks are owned by governments*.

**Proof of Theorem 1.** According to the information obtained from the method of calculating government shares and also using descriptive statistics, theorem 1 that the largest percentage of bank shares owned by the government is rejected, the average government shares in commercial banks of ten selected countries in the MENA It is equal to 33.97, which shows that the majority of shares in the MENA were not in the hands of politicians, but a large and effective percentage of shares were in the hands of governments. □

**Theorem 2.** Income smoothing is done in the banks of ten countries in the MENA region.

**Proof of Theorem 2.** Based on the model (1) of the research, we examined income smoothing in banks regardless of the percentage of government shares, and according to the results, income smoothing is done in banks and based on the significance of the whole model, there is a fundamental relationship between LLPs and bank income smoothing. So, theorem 2 is not rejecting. Also, we estimated model (1) for commercial banks in Iran, and based on the results, we observed that income smoothing has also been done in Iranian banks. □

**Theorem 3.** *Income smoothing occurs more in banks with a higher percentage of government shares than in banks with private ownership***.**

**Proof of Theorem 3.** In model (2), we entered the factor of government shares based on simple clustering (percentage of maximum shares more than 50% and less than 50%) and we observed that income smoothing takes place both in banks with maximum government shares and with fewer shares. So, in order to find out the impact of state ownership, we estimate another model. In model (3), the factor of government shares is considered based on HAC, and in the banks of the third group, which have the most government shares, income smoothing has been done more than in the first and second groups. According to the results of models (2) and (3) of this research, theorem 3 is not rejected. □

**Theorem 4.** *Government shareholders influence the smoothing income by using their political power.*

**Proof of Theorem 4.** In model (4), we added the election factor and examined it, and the results of this research show that income smoothing in banks with maximum government shares is more done in election years, which is a sign of graft of political power. In model (5), income smoothing in the election year is examined based on HAC and the results show that more income smoothing has been done in the election year. Next, to ensure the results of model (5), we examined one year before, one year after, and two years after the election, and the highest income smoothing in the year before the election is related to the group 3 banks that have the most government shares. Also, one year after the election, the percentage of income smoothing in two groups 2 and 1 was higher than one year before the election, which according to the political factor of the election, the results indicated the exercise of political power for personal purposes, which increased the income smoothing. However, in the two years after the election, the percentage of income smoothing has decreased, which shows the impact of politics and the political factor of the election on the management of bank resources. And all the results have shown that theorem 4 is not rejected that the policy factor influences income smoothing in government-controlled banks. □

## 5. Discussion

Doan et al. [41] defined income smoothing identification as the ratio of earnings before taxes and loan loss provisions to total assets. According to the results obtained from the data of this research, the coefficient of this ratio was positive and it indicated the smoothing of income through the ratio of LLPs to total assets in the banks of ten countries in the MENA.

From the banking control variables, we expected the coefficients related to the non-optional component of LLPs, i.e., the ratio of loan to total assets (L_i,j,t_) and the loan growth rate (ΔL_i,j,t_), to have positive coefficients [36]. According to the results of this research, in the (3) (4) and (5) models, in the group of banks that have the most government shares, these coefficients were positive.

For the other bank control variables, we expected the ratio of non-interest income to total assets (NONINT_i,j,t_) should have a positive coefficient. In models (1) and (4), this ratio was positive, in model (2) in banks with more than 50% government shares, and in models (3) and (5) in groups 3 and 2, which have a higher percentage of government shares, it was positive.

It is necessary to state that some of the coefficients examined in this article are positive and some others are negative. The positive coefficients of this research, such as EARN, which shows income smoothing, confirm the theory of income smoothing. The positive coefficients are placed in different groups, the more positive they are, they include the groups in which there are banks that have the most government shares. Also, coefficients have been examined in the research, which are negative, which means that banks have less possibility to smooth income in this situation.

We expected the coefficient of the demand deposits as a share of bank total deposits (DDEP_i,j,t_) to be negative [46, 61]. Demand deposits usually include saving accounts, checking accounts, and money market accounts that earn very little interest and less of which can be used in lending operations, and the owners of these deposits can withdraw their funds at any time, so the negative coefficient is due to Non-participation of these funds in lending operations. In models (1) (3) in groups 3 and 2, (4), and (5) in groups 1 and 2, this coefficient was negative. If it is positive, it means the violation of banks and the use of these accounts for lending operations.

We also expected that according to the results of Stiroh and Rumble [61] and Micco et al. [46], the coefficient of reporting total bank assets would be negative. It means, with the increase of bank assets, the level of income smoothing decreases, and the managers of these banks have less motivation and need for income smoothing. However, according to the results of this research in ten countries in the MENA, regardless of the percentage of government shares in the banks, with the increase in the bank’s assets, the bank managers were doing income smoothing more, and the coefficient of this variable was positive in all models. These results show the need for attention to the laws related to banks’ capital.

On the other hand, the coefficient of the lagged ratio of equity to total assets (EQUITY_i,j,t-1_) was predicted based on the results of Stiroh and Rumble [61] and Micco et al. [62] to be negative Managers should save these funds for the development and improvement of the bank and prevent bankruptcy. The positiveness of this coefficient means that managers use these funds for other purposes. According to the data results of this research, in the models (1) (2) (3) in groups, 1 and 3, model (4) in banks with state shares of more than 50%, and model (5) this coefficient was negative and has no effect on LLPs.

If the three variables related to Bank Regulation including overall capital stringency, bank accounting informative, and official supervisory power of banks have negative coefficients, it means the correct and strong implementation of regulatory laws and regulations [31,37,54,71]. But the positiveness of these coefficients indicates the need for better supervision, and according to the results of this research in ten MENA countries, it is necessary to strengthen the rules and regulations on banking operations.

Regarding macro control variables, if the coefficient of REALGDP denotes the log of Gross Domestic Product (GDP) per capita, while is positive, it indicates the positive impact of banks on the development of the country, and the negative coefficient indicates that banks do not have a positive impact on GDP [41]. According to the results of the research data in the model (1), this coefficient is positive and it can be concluded that in general and during the years 2015–2019, the activities of the banks of ten countries in the MENA have led to the development of these countries. In models ()2 ()3 banks with more than 50% government shares, model (4) both groups, model (5) groups 3 and 2, this coefficient was negative. Considering that models (4) and (5) in The election year have been estimated and the results indicate the negative impact of the political factor on the banking activity and as a result the development of the countries. In the year before the election, group 3, which includes banks with the highest percentage of government shares, had a negative coefficient, in one year after the election Also, two groups of 3 and 1 had a negative coefficient, and in the two years after the election, this coefficient was positive in all three groups, which is another confirmation of the negative effect of government shareholding and political factors on the activity of banks.

The positivity of the INFLATION coefficient refers to the deflated Consumer Price Index (CPI) for each country with the base year of 2010. It means an increase in inflation as a result of banking operations [41]. This macro control variable in model (1) of the research, which is related to the general state for 5 years, and model (5) for two years after the election in groups 3 and 2, has a negative coefficient and indicates the reverse effect of banking operations on the inflation rate and causes Inflation has decreased. In models (2) (3) (4), and (5) in two groups 1 and 3, the estimate of the model (5) for one year before the election and one year after the election in all three groups is positive and shows a negative impact of government shareholding and the political factor of the election is on banking operations and as a result, the increase in inflation.

## 6. Conclusions and future research directions

Based on the model to obtain the percentage of government shares, we obtained the average percentage of government shares of 33.97% for the commercial banks of ten countries in MENA. According to the estimate of the model (1), income smoothing has been done in commercial banks of ten countries in MENA during the years 2015–2019. According to the results of models (2) and (3), income smoothing in commercial banks of ten countries in MENA has been done more in banks with a higher percentage of government shares. According to models (4) and (5), the political factor of the election has increased income smoothing. By estimating model (5) for one year before, one year after, and two years after the election, it was found that in one year before the election, group 3, which had the highest percentage of government shares, experienced the highest of income smoothing, but for groups 1 and 2, which include banks with a lower percentage of government shares, have had the least leveling of income in one year before the election. One year after the election, income smoothing for groups 1 and 2 has been done in less than one year before the election. For group 3, in one year after the election, the income smoothing has been done lower than in the year of the election. The effects of the political factor of the election have almost disappeared in the two years after the election, and the income smoothing done this year has decreased for all three groups. From all the results obtained from the data of this research, it can be concluded that the policy has had an impact on income smoothing in commercial banks under the control of the government of ten countries in MENA during the years 2015–2019. One of the most important limitations of this research is that it was difficult to find a match between macroeconomic policies and elections in these countries, and many policies had no special relationship with elections. It was also not possible to access the information of a number of commercial banks active in MENA countries, especially the commercial banks of less developed countries. This is due to the fact that there is no suitable database for providing information and financial statements of these banks. For future research, it is recommended to explore income smoothing practices using loan loss provisions in banks located outside the MENA region, such as those in East Asian countries. Additionally, to enhance the stability of bank performance and mitigate imbalances in balance sheets, the application of advanced analytical models, such as data envelopment analysis and its variants, can be considered [72–86].

Despite the robustness of our empirical strategy, several limitations should be acknowledged. First, while we employ the system-GMM estimator to address endogeneity and dynamics in the model, one potential limitation is that certain explanatory variables, such as government ownership, may exhibit limited within-bank time variation during the sample period. Although we mitigate this through interaction terms and clustering approaches, this limitation may affect identification strength. Second, although we report standard diagnostic tests such as the Hansen and AR(2) tests, we acknowledge that GMM estimation relies on instrument validity, which can be sensitive to model specification. Third, while election-year data were primarily drawn from authoritative databases, in some cases we had to rely on publicly available official websites and media sources, which may introduce some measurement inconsistency across countries. Lastly, due to data constraints, we were unable to fully decompose loan loss provisions into discretionary and non-discretionary components across all banks, which may affect the precision of our income smoothing proxy. Future research may address these limitations by utilizing alternative identification strategies such as difference-in-differences, or by incorporating more granular ownership and political data.
